# Dynamic Regulation of the Molecular Mechanisms of Regulatory T Cell Migration in Inflamed Skin

**DOI:** 10.3389/fimmu.2021.655499

**Published:** 2021-05-10

**Authors:** M. Ursula Norman, Zachary Chow, Sarah L. Snelgrove, Peemapat Prakongtham, Michael J. Hickey

**Affiliations:** Centre for Inflammatory Diseases, Department of Medicine, Monash Medical Centre, Monash University, Clayton, VIC, Australia

**Keywords:** regulatory T cell, skin, inflammation, migration, α_v_ integrin, PI3 kinase

## Abstract

The presence of regulatory T cells (Tregs) in skin is important in controlling inflammatory responses in this peripheral tissue. Uninflamed skin contains a population of relatively immotile Tregs often located in clusters around hair follicles. Inflammation induces a significant increase both in the abundance of Tregs within the dermis, and in the proportion of Tregs that are highly migratory. The molecular mechanisms underpinning Treg migration in the dermis are unclear. In this study we used multiphoton intravital microscopy to examine the role of RGD-binding integrins and signalling through phosphoinositide 3-kinase P110δ (PI3K p110δ) in intradermal Treg migration in resting and inflamed skin. We found that inflammation induced Treg migration was dependent on RGD-binding integrins in a context-dependent manner. α_v_ integrin was important for Treg migration 24 hours after induction of inflammation, but contributed to Treg retention at 48 hours, while β_1_ integrin played a role in Treg retention at the later time point but not during the peak of inflammation. In contrast, inhibition of signalling through PI3K p110δ reduced Treg migration throughout the entire inflammatory response, and also in the absence of inflammation. Together these observations demonstrate that the molecular mechanisms controlling intradermal Treg migration vary markedly according to the phase of the inflammatory response.

## Introduction

CD4^+^ regulatory T cells (Tregs) are recognized for their capacity to control inappropriate activity of the immune system both systemically, and in peripheral sites (1–3). While Tregs have powerful systemic effects, in many cases suppression of inflammation in the periphery is dependent on the local actions of Tregs in these sites. A growing body of evidence indicates that the actions of these cells are of particular relevance in the skin. A high proportion of circulating Tregs express adhesion molecules and chemokine receptors used by immune cells for skin entry (4, 5). Also, healthy skin in humans and mice contains numerous Tregs, predominantly restricted to the dermis (6–9). In patients affected by Treg dysfunction, the skin is one of the organs severely affected by uncontrolled inflammation (10). Moreover, in models of skin inflammation, Tregs accumulate in the skin and selective inhibition of their homing to, or retention in, the skin results in uncontrolled skin inflammation (1, 2, 4, 6, 11, 12). Despite this, little is known about the behaviour of Tregs within the skin. We have previously used intravital multiphoton microscopy (MP-IVM) to demonstrate that in the absence of inflammation, most Tregs are static, but that upon induction of inflammation, many Tregs become highly migratory. Notably, Treg migration remains increased after the inflammatory response has resolved (6, 13). While studies of conventional effector CD4^+^ T cells in models of skin infection have demonstrated that efficient intradermal migration is critical for effective anti-microbial function (14), the requirement for Treg migration throughout the skin in resolution of skin inflammation is unclear. To test this idea, it is first necessary to understand the molecular basis of this response.

Interstitial immune cell migration is controlled by cell surface molecules as well as intracellular molecular pathways. Previous intravital imaging studies of effector Th1 and Th2 CD4^+^ T cells in the skin demonstrated a key role for the Arg-Gly-Asp (RGD)-binding α_v_ integrin, partnered with either the β_1_ or β_3_ integrin, in facilitating their migration in the inflamed dermis (14, 15). Whether Tregs, as a CD4^+^ subset, also utilize RGD-binding integrins for motility in inflamed skin is unknown. The α_v_ integrin is highly expressed on Tregs in inflamed tissues (16). However, investigation of its contribution to Treg accumulation in inflammatory responses in the brain and colon has shown divergent results. Absence of the α_v_ integrin impaired the capacity of Tregs to accumulate in the colon and reverse colonic inflammation, while in the brain this integrin was not required for Treg accumulation or anti-inflammatory function (16). The role of the RGD-binding integrins in Treg migration in skin is yet to be explored.

Treg motility and localisation can also be influenced by intracellular signalling pathways activated via the T cell receptor (TCR), particularly those of the phosphoinositide 3-kinase (PI3K) lipid kinase family. The PI3K p110δ subunit activated in response to TCR-mediated antigen recognition, co-stimulatory CD28 receptor ligation and ICOS-ICOS ligand interactions, plays numerous roles in T cells, including in promotion of migration (17–19). In Tregs, PI3K p110δ is critical for Treg suppressor function and peripheral tissue homing (20–23). Inhibition of PI3K p110δ also reduces the recruitment of Tregs into tissue driven by recognition of cognate antigen presented by endothelial cells (24). However, the contribution of this signalling pathway to interstitial migration of Tregs following their entry into tissue is unknown. Therefore, the overall aim of this study was to investigate the molecular mechanisms that control Treg interstitial migration in the skin. Using MP-IVM, we found that RGD-binding integrins contributed to intradermal Treg migration to variable degrees, according to the phase of the inflammatory response, while signalling through the PI3K p110δ subunit was important for Treg motility under both resting and inflamed conditions.

## Materials and Methods

### Mice

Experiments were conducted using eight- to 12-week-old male B6N.129(Cg)-Foxp3tm3Ayr/J (Foxp3-GFP) mice, were bred in-house under specific pathogen-free conditions. All animal experiments were approved in advance by the Monash Medical Centre Animal Ethics Committee B.

### Antibodies and Reagents

All antibodies were purchased from either BD Biosciences or Biolegend. For analysis of integrin expression on leukocytes extracted from skin samples or draining lymph nodes (dLNs), cells were stained with a Live/Dead Fixable Near-IR Dead Cell Stain Kit (Invitrogen) and anti-CD45 (clone 30-F11), anti-CD4 (clone GK1.5), anti-CD8 (clone 53-6.7), anti-CD103 (clone M290), anti-α_V_ integrin (clone HMa5-1 and RMV-7), anti-β_1_ integrin (clone HMb1−1); anti-β_3_ integrin (clone 2C9.G2), anti-ICOS (clone 7E.17G9), anti-CD69 (clone), anti-CD62L (clone MEL-14) and anti-CD44 (clone IM7). For *in vivo* function-blocking experiments, 25 µg of the following azide-free, low endotoxin, antibodies (all from Biolegend) were injected intradermally 2 hours prior to imaging: anti-α_V_ (clone HMa5-1) and anti-β_1_ (clone HMb1-1, 5 x 10 µL in a 10 x 20 mm region of skin). Control mice received the same amount of non-specific isotype control antibodies (Armenian Hamster IgG).

### Oxazolone Induced Model of Contact Sensitivity

Oxazolone-induced contact hypersensitivity (CS) inflammatory response was induced as previously described (6, 13, 25). To initiate CS, mice were sensitized by application of 50 µL of 5% oxazolone (Sigma-Aldrich, St Louis, MO) dissolved in acetone/olive oil (4:1) to a shaved area on the back. Five to seven days later, mice were challenged with 50 µL of a 1% oxazolone/acetone/olive oil solution to a shaved area (1 x 2 cm) on the right abdominal flank skin. Multiphoton imaging was performed in untreated mice as well as at 24, 48, 72 hours (h) and 6 days post challenge.

### IC-87114 Treatment

The selective p110δ inhibitor, IC-87114 (Santa Cruz Biotech) was injected i.p. (15 mg/kg in 10% DMSO in sterile saline) into either untreated, irritant-treated, or 24 h and 48 h CS-challenged mice (17). Control mice received 10% DMSO in saline.

### RGD Peptide Treatment

Two hours prior to imaging, mice were injected intradermally with RGD or control RAD peptide (50 µg, Mimotopes Pty Ltd) in 50 µL sterile saline (5 x 10 µL injections) in flank skin.

### Innate Skin Inflammation

Two models of innate skin inflammation were examined. The innate inflammatory response to hapten was examined by application of 1% oxazolone (in acetone/olive oil vehicle) to the flank skin of naïve Foxp3-GFP mice and MP-IVM performed at 24 hours post challenge. Alternatively, inflammation was induced using Croton oil (CO). CO-induced skin inflammation was initiated by application of 50 μL of 2% CO oil (v/v in acetone) to a shaved area (1 x 2 cm) of the abdominal flank (26, 27). As a measure of inflammation in these experiments, ear swelling was assessed following application of 20 µL of 2% CO in acetone vehicle to one ear and 20 µL of vehicle alone to the contralateral ear. Ear thickness was measured using a micrometer and swelling expressed as the difference between challenged and control ears.

### Multiphoton Microscopy of the Flank Skin

Flank skin was prepared for multiphoton microscopy as previously described (6). Briefly, mice were anesthetized by i.p. injection of 150 mg/kg ketamine hydrochloride (Troy Laboratories, Smithfield, NSW, Australia) and 10 mg/kg xylazine (Pfizer, West Ryde, NSW, Australia) and the right jugular vein cannulated for the administration of further anesthetic. The body temperature of the mice was maintained by a heat pad. The hair from the previously shaved, challenged area of flank skin was further removed by brief treatment with depilatory cream (Nair). A midline incision was made in the abdominal skin and the flank skin extended over a heated pedestal with the epidermal side facing up. The exposed area was immersed in saline and enclosed with coverslip held in place by vacuum grease.

Skin MP-IVM was performed with an Olympus FVMPE-RS microscope equipped with a 25 x 1.05 NA lens, four non-descanned detectors and a multiphoton laser (Insight X3, *Spectra Physics*). Experiments were performed at 900 nm excitation. Images were collected at a resolution of 512 x 512 pixels, 2 µm z step size, to depths of 100-150 µm, acquired every 60 seconds for a total of 30 minutes per recording. Typical experiments involved 3 recordings of non-overlapping regions of the flank skin.

### Image Analysis

Treg behaviour was analysed using *Imaris* image analysis software (Bitplane, Zurich, Switzerland). Treg abundance was calculated by counting Tregs in 2-3 randomly-selected regions of the dermis. Data were averaged and expressed as cells/mm^3^. Migration was tracked using the surfaces tool applied to individual Tregs and track displacement (µm), mean velocity (µm/min) and confinement (displacement divided by the total track length) determined. In order to qualitatively describe Treg migratory behaviour, mean velocity for individual cells was plotted against confinement as previously described (28). This plot is subdivided into quadrants based on a threshold migration velocity of 2 µm/min, which defines cells that move more than one cell width from their point of origin during the period of observation, and a threshold confinement ratio of 0.2 which characterizes cells more likely to migrate in a directed manner (28). Dermal collagen fibre density, as determined from second harmonic generation (SHG) signal intensity, was examined at a single time point extracted from the same recordings, as previously described (14). Images were cropped to a 150 µm x 150 µm region of the image stack to exclude hair follicles and SHG signal in captured images analysed using the FIJI image analysis software determining the average SHG signal intensity.

### Flow Cytometric Analysis of Skin Tregs

To analyse immune cells from abdominal skin, flank skin of untreated or challenged (24 or 48 h CS) Foxp3-GFP mice was shaved and cleaned with 70% ethanol. A 1 x 2 cm region of skin was excised and placed into a Liberase TL (0.25 mg/mL, Roche)/DNase I recombinant, RNase-free (0.5 mg/mL, Roche) in RPMI 1640 (Gibco Cell Culture Media) 10% FCS solution. Skin was incubated for 60-90 minutes at 37°C in a cell culture incubator (5% CO_2_). EDTA (25 mM) was added to stop the digestion and the skin gently macerated with scissors. Digested tissue was washed 2x with complete RPMI and filtered sequentially through 70 µm and 40 µm EASYstrainer cell strainers (Greiner Bio-One International) to generate a single cell suspension. All cell staining was completed in MACs buffer (0.5% BSA, 2 mM EDTA in PBS). For flow cytometry, cells were analysed on a BD LSR Fortessa X-20 and data analysed with FlowJo software.

### Statistics

GraphPad Prism software was used for all statistical analysis. Unpaired 2-tailed *t* tests (Mann-Whitney) were used for comparison of groups, while one-way ANOVA (Kruskal-Wallis tests with Dunn’s test correction for multiple comparisons) was used to compare three or more groups.

## Results

### During CS, Tregs in Flank Skin Dermis Are Predominantly Found in Persistent Clusters of Highly Migratory Cells

**Figure 1:**
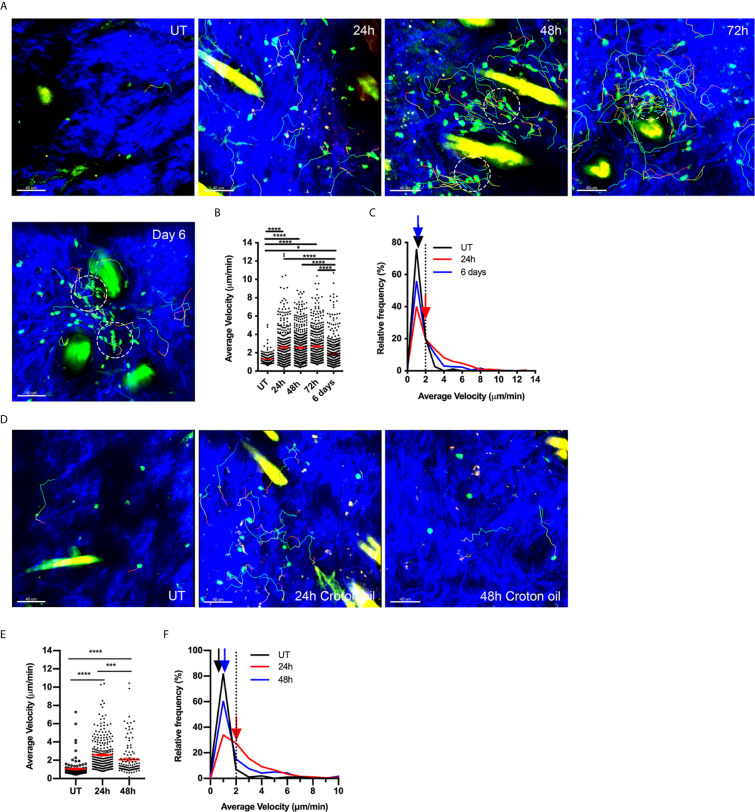
Tregs increase migration around hair follicles in response to both adaptive and innate stimuli. **(A–C)** Effects of CS response on Treg migration. Treg motility in the flank skin was examined in Foxp3-GFP mice using MP-IVM in either untreated (UT) skin, or over a time course of CS-induced inflammation. **(A)** MP-IVM images of the dermis of Foxp3-GFP mice showing the paths of migration (multicoloured) of GFP^+^ Tregs (green) in UT mice, and CS mice 24, 48, 72 h and 6 days after challenge. Scale bars - 40 µm. Collagen fibres (blue) are visualized by secondary harmonic signal (SHG). Dotted circles denote intradermal clusters of Tregs. **(B)** Alterations in Treg migration velocity over a time course of CS. Data show velocities for individual Tregs as well as group mean ± sem. **(C)** Relative frequency distribution of the average velocity of individual Tregs from UT, 24 h or 6 days post CS challenge. Arrows indicate the median velocity, and the dotted line indicates the 2 µm/min motility threshold. **(D–F)** Treg migratory response in innate skin inflammation induced by Croton oil (CO). **(D)** MP-IVM images of intradermal Treg migration paths in Foxp3-GFP mice undergoing CO-induced inflammation, showing example images from untreated skin (UT), and 24 & 48 h post CO application. Scale bars - 40 µm **(E)** Alterations in Treg migration velocity during CO-induced inflammation. Data show velocities for individual Tregs as well as group mean ± sem. **(F)** Relative frequency distribution of the average velocity of individual Tregs from UT, 24 or 48 h CO-treated mice. Arrows indicate the median velocity, and the dotted line indicates the 2 µm/min motility threshold. All data are shown as mean ± SEM from 3-6 mice/group. Data were analysed using a Kruskal-Wallis test with Dunn’s multiple comparison tests between all timepoints. *p < 0.05, ***p < 0.001, ****p < 0.0001 for comparisons shown.

We have previously used MP-IVM of flank skin of Foxp3-GFP mice to show that Tregs in the skin are mostly non-motile and located in the dermis adjacent to hair follicles (6). Induction of CS leads to increased Treg abundance in the skin as well as an increase in the proportion of Tregs undergoing migration (6). In the present study, we examined this response in greater detail, demonstrating that the increase in migratory behaviour extends to at least 3 days post challenge, with Treg migration returning to basal levels by 6 days post-challenge (**Figures 1A, B**, **Supplementary Videos 1, 2**). The proportion of motile Tregs, defined as having an average speed ≥ 2 µm/min, increased from 8.5% in untreated skin to greater than 50% 24 hours post challenge (**Figure 1C**). This increase in motility was maintained through to 72 hours (**Figure 1B**). The majority of the Tregs migrated within a 50 µm radius of hair follicles and sometimes traversed completely around the follicle. Small clusters of Tregs around hair follicles were already apparent 24 hours post CS challenge (**Figure 1A**, panel 2) and these became more prevalent and more densely packed with cells 48 and 72 hours post CS challenge (**Figure 1A**, panels 2-4). These clusters were highly dynamic with Tregs migrating into, within and out of these areas (**Supplementary Videos 1, 2**), in behaviour similar to what has been previously described for HSV-specific CD4^+^ T cells in virus-infected skin (29). Six days after CS challenge, Tregs were still present in diffuse clusters surrounding hair follicles but the majority of these Tregs were not motile (70% immotile – **Figure 1C**).

To assess whether this persistence in Treg migration also occurs in an innate form of inflammation, we examined intradermal Treg migration in skin challenged with CO. CO application to flank skin induces an irritant response characterised by neutrophil and monocyte influx (30). Here we observed that CO induced a significant increase in Treg motility within the skin within 24 hours (**Figures 1D–F**). However, this behaviour did not persist for as long as in CS, as by 48 hours post CO application, the velocity and proportion of motile Tregs were approaching levels in untreated skin (**Figures 1E, F**), despite inflammation, as shown by ear swelling, persisting at this time point (**Supplementary Figure 1A**). Moreover, CO did not induce Treg clustering (**Figure 1D**) or recruitment of Tregs into the skin (**Supplementary Figure 1B**). Together these findings indicate that antigen-driven inflammation induces persistent changes in intradermal Treg migration and clustering while the effects of an innate stimulus on Treg motility are more subtle and less prolonged.

### CS Alters the Distribution of Dermal Collagen

**Figure 2:**
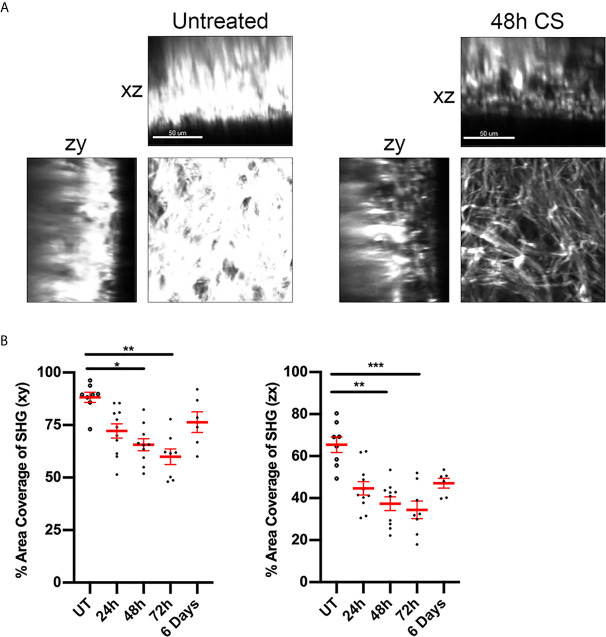
CS alters ECM fibre distribution in flank skin. The effect of CS-induced inflammation on the distribution of dermal collagen fibres was assessed via SHG in MP-IVM experiments. **(A)** Example images of collagen fibre distribution in untreated skin and at 48 h post CS induction. Images represent single planes from 3D volumes in either the planar (xy), zx or zy planes. Scale bar - 50 µm. **(B)** Percentage of area filled by SHG signal in or the xy or zx cross sections, taken 60 µm below the skin surface. Data are shown for individual mice and as mean ± SEM from 3-7 mice per group. Data were analysed using Kruskal-Wallis test with Dunn’s multiple comparison. *p < 0.05, **p < 0.01, ***p < 0.001 for comparisons shown.

Inflammation of the skin is associated with changes to extracellular matrix (ECM) composition and structure, with decreases in the density of connective fibres associated with increased CD4^+^ T cell motility within the dermis (14). The intensity of the SHG signal arising from multiphoton imaging can be used to assess changes in the organisation of fibrillar collagen during inflammation (31). Here we used this approach to detect changes in dermal collagen during the CS response. In untreated skin, the SHG signal had a dense mesh-like appearance and covered ~85% of a 150 x150 µm area (xy) of skin (**Figures 2A, B**). CS-induced inflammation induced changes in collagen organisation, with the fibres being less organised and more loosely-packed. These changes were reflected in a significant reduction in the percentage of area positive for SHG signal over the course of the response, with the lowest signal being observed 48-72 hours post-challenge (**Figure 2B**). Similar reductions in SHG intensity were observed in the zx cross-sectional skin area. By six days, SHG signal intensity was not significantly different to that seen in untreated skin. These experiments reveal the time course of inflammatory changes in flank dermal collagen during the CS model.

### The Role of RGD-Binding Integrins in Treg Motility Varies During Contact Sensitivity

**Figure 3:**
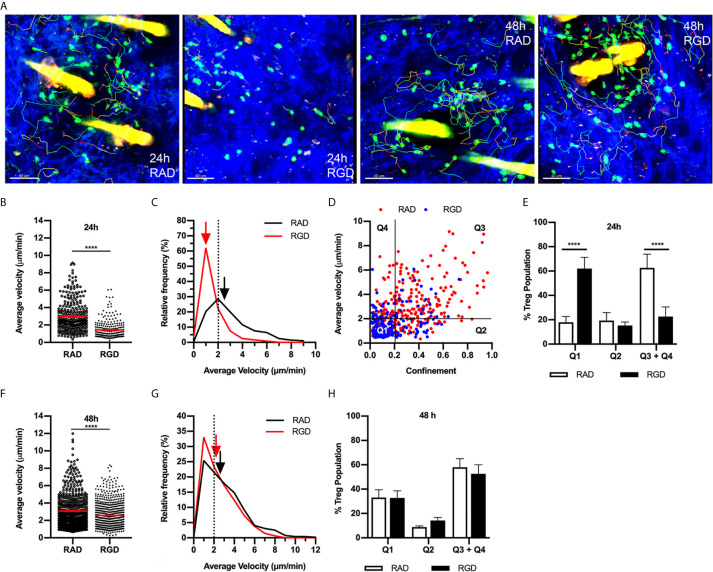
RGD peptide-binding integrins modulate Treg migration during CS. The effect of RGD-binding integrins on Treg migration in CS-inflamed skin was assessed using MP-IVM. Treg migration was examined following i.d. injection of RGD or RAD control peptides 2 h prior to visualization by MP-IVM. **(A)** MP-IVM images of Treg migration paths in RAD- and RGD-treated Foxp3-GFP mice 24 and 48 h post-CS induction. **(B–H)** Treg migration in mice treated with either RAD or RGD peptides 24 or 48 h after induction of CS. **(B, F)** Average velocity of Treg migration 24 **(B)** and 48 h **(F)** after induction of CS. Data show velocities for individual Tregs as well as group mean ± sem. **(C, G)** Relative frequency distributions of the average velocities of individual Tregs from RAD- or RGD-treated mice 24 **(C)** and 48 h **(G)** post CS induction. Arrows indicate the median velocity, and the dotted line indicates the 2 µm/min motility threshold. **(D)** Dot plot distribution of individual Tregs from RAD (red) and RGD (blue)-treated mice at the 24 h CS timepoint, plotted according to average velocity and confinement. **(E, H)** Distributions of Tregs within the four quadrants plotted for the **(E)** 24 h and **(H)** 48 h CS timepoints. Data represent the mean ± SEM of > 250 cells derived from 3-5 mice/group. Quadrant data represent mean ± SEM of data averaged from 3 fields of view (fov)/mouse. Velocity data in **(B, F)** were compared using Mann-Whitney tests. Quadrant data in **(E, H)** were assessed using a multiple comparison *t* test using the Holm-Sidak method (α, 0.05). ****p < 0.0001 for the comparisons shown.

Migration of effector CD4^+^ T cells in the inflamed dermis has been shown to be dependent on integrins that bind the RGD motif in fibronectin (14, 32). Moreover, inflammation-induced changes in the structure of dermal collagen correlate with increased levels of fibronectin closely associated with collagen fibres in the skin (32). Therefore, we examined the role of RGD-binding integrins in Treg dermal interstitial migration 24 and 48 hours post CS initiation (**Figures 3A–H**). Intradermal administration of RGD peptide, which inhibits integrin binding to fibronectin, resulted in the almost complete cessation of Treg migration 24 hours post CS challenge, a response not seen in mice treated with the control RAD peptide (**Figures 3A, B**, **Supplementary Video 3**). This was seen as a 2-fold reduction in average Treg migration speed (RGD - 1.4 ± 0.1 µm/min, RAD - 3.0 ± 0.1 µm/min) (**Figure 3B**), and a shift in the relative frequencies of Treg velocity with 65% of the Treg population in RGD peptide-treated mice displaying speeds below 2 µm/min compared with 20% in RAD peptide-treated mice (**Figure 3C**). Similarly, the average Treg displacement was significantly reduced (RGD - 7.4 ± 0.7 µm vs. RAD - 25.0 ± 1.4 µm, *p*<0.0001). This reduction meant that most Tregs in the RGD peptide-treated mice did not move further than one cell distance from their starting position.

This behaviour was further characterised by plotting the average velocity of individual Tregs against their confinement ratio (straight-line migration distance divided by total track length) (**Figure 3D**). Using the definition of a migratory Treg as having an average velocity of ≥ 2 µm/min and using a threshold confinement value of 0.2 allows this plot to be subdivided into quadrants that allow qualitative description of different Treg behaviours. Here quadrant 1 (Q1) represents cells that are static (low velocity/low confinement), quadrant 2 (Q2) represents Tregs that display intermittent migration such that their average velocity remains below 2 µm/min, while quadrant 3 (Q3) represents Tregs that display higher velocities and confinement ratios which equate to continuous migration. Finally, quadrant 4 (Q4) represents cells with high migration velocity, but low confinement such that they remain relatively close to their site of origin (**Figure 3D**). 24 hours post CS, the majority of the Treg population (60%) were in Q3 + Q4, with approximately 20% found in each of Q1 and Q2. At 24 hours CS, treatment with RGD peptide shifted the Treg population from being highly migratory (Q3+Q4) to static (Q1) with no changes to the intermittent migratory population (Q2) (**Figure 3E**). In contrast, 48 hours post challenge, the impact of RGD peptide treatment was more subtle, inducing a small but significant decrease in Treg average velocity (**Figures 3F, G** and **Supplementary Video 3**) and average displacement (RGD - 20.2 ± 1.0 µm vs. RAD - 24.0 ± 1.1 µm, *p*<0.005). However, this alteration did not change the percentages of Tregs within the different quadrants of the velocity versus confinement plots (**Figure 3H**). These findings indicate that RGD-binding integrins are critical for Treg migration 24 hours post CS induction, but contribute less to Treg motility at 48 hours.

### α_v_ but Not β_1_ Integrin Contributes to Treg Motility During the CS Response

**Figure 4:**
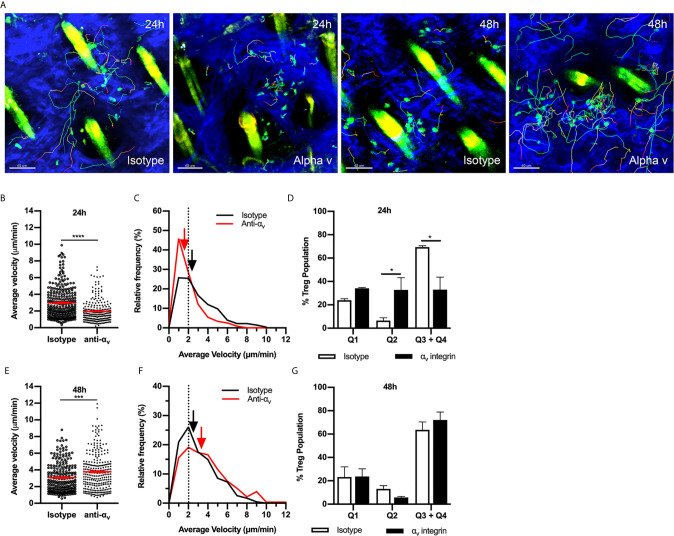
α_v_ integrin has a variable role in Treg migration during CS-induced inflammation. The contribution of the α_v_ integrin to Treg migration in CS-inflamed skin was assessed using MP-IVM of CS-inflamed skin, following administration of anti-α_v_ integrin or isotype control antibodies 2 h prior to visualization by MP-IVM. **(A)** MP-IVM images of Treg migration paths in Foxp3-GFP mice treated with anti-α_v_ integrin or isotype control antibody at the 24 or 48 h CS timepoints. **(B–G)** Effect of anti-α_v_ integrin treatment on Treg migration velocity 24 **(B)** or 48 h **(E)** after induction of CS. Data show velocities for individual Tregs as well as group mean ± sem. **(C, F)** Relative frequency distributions of the average velocities of individual Tregs from anti-α_v_ integrin and isotype control antibody-treated mice, shown for 24 **(C)** and 48 h **(F)** post-CS induction. Arrows indicate the median velocity, and the dotted line indicates the 2 µm/min motility threshold. **(D, G)** Distributions of Tregs within the four quadrants of the velocity vs. confinement plots shown for 24 h **(D)** and 48 h **(G)** post induction of CS. Data represent the mean ± SEM of > 230 cells derived from 3-4 mice/group. Quadrant data represent mean ± SEM of data averaged from 3 fov/mouse. Data in **(B, E)** were analysed using Mann-Whitney test. ***p < 0.001, ****p < 0.0001. Quadrant data in **(D, G)** were compared using a multiple comparison *t* test using the Holm-Sidak method (alpha 0.05), *p < 0.05 for the comparisons shown.

We next investigated potential RGD-binding integrins responsible for intradermal Treg migration during CS, focusing on the α_v_ integrin found previously to be critical for effector CD4^+^ T cell migration in inflamed skin (14). α_v_ integrin inhibition at 24 hours CS resulted in significant decreases in Treg average velocity (**Figures 4A, B**, **Supplementary Video 4**) and displacement (anti-α_v_ - 17.8 ± 1.6 µm vs. isotype control - 23.8 ± 1.9, *p*<0.05). The decrease in average velocity was due to a shift in the relative frequency velocity curve with 48% of the Tregs having an average velocity < 2 µm/min compared to 26% in the isotype control group (**Figure 4C**). Assessment of velocity versus confinement revealed that anti-α_v_ antibody caused a significant reduction in the percentage of Tregs within the highly migratory (Q3+Q4) populations, shifting to the intermittently migratory population (Q2) (**Figure 4D**). This effect differed from that seen with RGD peptide administration, which caused an increase in the static population at 24 hours CS (**Figure 3E**). The effect of anti-α_v_ integrin antibody treatment at 48 hours was markedly different from that seen at 24 hours. 48 hours post CS challenge, anti-α_v_ integrin resulted in a subtle but significant increase in Treg velocity (**Figures 4E, F** and **Supplementary Video 4**) and total track length (anti-α_v_ – 82.8 ± 2.9 µm *vs*. isotype control – 69.6 ± 2.5 µm, *p*<0.005) but not track displacement (data not shown). However, these changes did not change the distribution of cells in the different modes of migratory behaviour (**Figure 4G**). Taken together these findings indicate that the α_v_ integrin is important for Treg directional migration at 24 hours but not at 48 hours post CS challenge.

**Figure 5:**
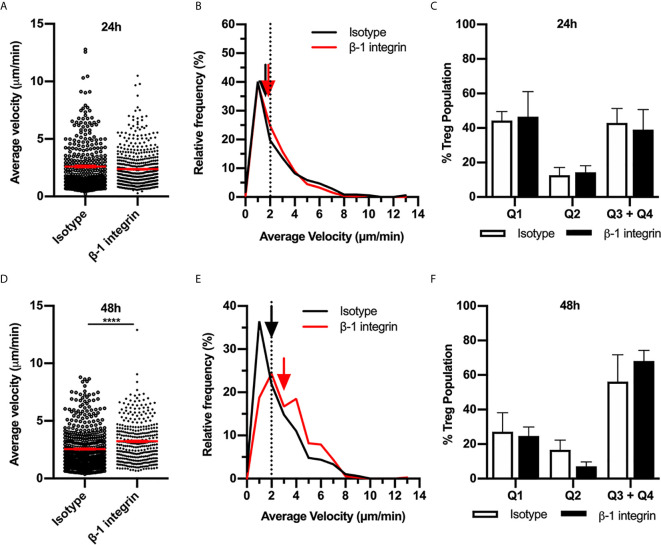
β_1_ integrin modulates Treg migration speed during 48 h CS-induced inflammation. The contribution of the β_1_ integrin to Treg migration in CS-inflamed skin was assessed using MP-IVM of CS-inflamed skin following the i.d. injection of anti-β_1_ integrin or isotype control antibodies 2 h prior to visualization by MP-IVM. Effect of anti-β_1_ integrin treatment on Treg migration velocity 24 h **(A–C)** or 48 h **(D–F)** after induction of CS. **(A, D)** Treg migration velocity 24 **(A)** and 48 h **(D)** post CS induction. Data show velocities for individual Tregs as well as group mean ± sem. **(B, E)** Relative frequency distributions of the average velocities of individual Tregs from 24 h **(B)** or 48 h **(E)** after CS induction. Arrows indicate the median velocity and dotted line indicates the 2 µm/min threshold of motility. **(C, F)** Distributions of Tregs within the four quadrants of the velocity *vs*. confinement plots shown for 24 h **(C)** and 48 h **(F)** post induction of CS, in anti-β_1_ integrin *vs*. isotype control mice. Data represent the mean ± SEM of > 350 cells derived from 3-5 mice/group. Quadrant data represent mean ± SEM of data averaged from 3 fov/mouse. Data in **(A, D)** analysed using Mann-Whitney test. ****p < 0.0001.

To assess the possibility that other ECM-binding integrins contribute to Treg migration, we investigated the role of the β_1_ ****integrin in Treg migration during the CS response. In addition to α_v_ integrin, β_1_ ****integrin partners with the α chains 1-10, therefore, inhibiting this integrin would interfere with binding to most other ECM molecules in addition to RGD-containing components. 24 hours post CS induction, inhibition of β_1_ ****integrin antibody did not alter Treg behaviour (**Figures 5A–C**). In contrast at 48 hours, β_1_ ****integrin inhibition significantly increased Treg velocity (**Figures 5D, E**), similar to the effect of α_v_ integrin inhibition at the same time point. However, this increase in average speed was not due to a difference in the percentages of Treg within different quadrants of the velocity vs. confinement plots (**Figure 5F**). Overall, this indicates that during the early CS response (24 h), α_v_ integrin likely interacts with β_3_ ****integrin rather than β_1_ ****integrin to direct Treg migration, while at 48 hours CS, α_v_ together with β_1_ integrin acts to inhibit Treg movement.

**Figure 6:**
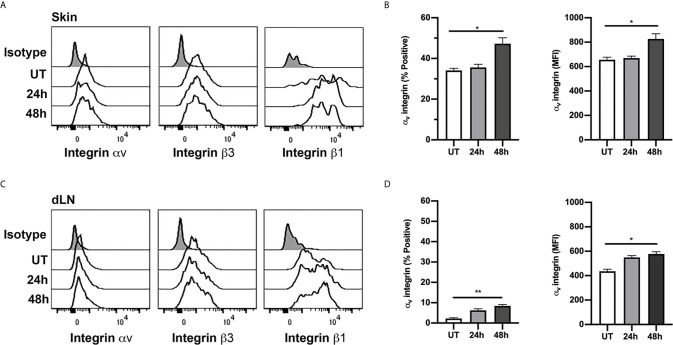
Expression of α_v_, β_1_ and β_3_ integrins on Tregs in skin and dLN. α_v_, β_1_ and β_3_ integrin expression on Tregs from skin and draining lymph nodes (dLN) of untreated (UT) mice, and 24 and 48 h post CS induction was assessed using flow cytometry. **(A, C)** Example histograms of Tregs from skin **(A)** and dLN **(C)** comparing expression of α_v_, β_1_ and β_3_ integrins. Data also shown for isotype control (shaded histograms). **(B, D)** ⍺_v_ integrin expression on Tregs from skin **(B)** and dLN **(D)**, shown as % positive and mean fluorescence intensity (MFI) on cells from untreated (UT) mice, and 24 and 48 h post CS induction. Data are shown as mean ± SEM derived from 4 mice/group. Data were evaluated via Kruskal-Wallis test with Dunn’s multiple comparison tests between all timepoints. *p < 0.05, **p < 0.01 for the comparisons shown.

One possible explanation for differences in the functional roles of RGD-binding integrins at different CS timepoints is that Tregs may alter expression of the relevant integrins during the response. To investigate this possibility, we used flow cytometry to examine expression of α_v_, β_1_ and β_3_ integrins on Tregs isolated from flank skin at different stages of the CS response (**Figures 6A, B**). In uninflamed skin, ~34% of Tregs expressed α_v_ integrin. By comparison, splenic Tregs in non-inflamed mice were comparatively low for α_v_ integrin (10.1 ± 2.1% α _v_^+^, mean ± sem, n=4), indicating that elevated α_v_ integrin phenotype is characteristic of Tregs in the skin, and not dependent on inflammatory activation. The proportion of α_v_^+^ Tregs in the skin was unaltered at 24 hours CS, but significantly elevated to ~ 47% at 48 hours after CS induction (**Figures 6A, B**), with this increase also apparent as an increase in the average α_v_ integrin staining intensity (**Figure 6B**). In contrast, in skin dLNs, α_v_^+^ Tregs were very low in abundance under resting conditions (~2% of Tregs), although this increased during CS, being significantly increased, in proportion and expression level, by 48 hours (**Figures 6C, D**). The β_1_ ****integrin was expressed at high levels on most skin-derived and dLN Tregs, with no significant differences in expression between uninflamed and CS mice (**Supplementary Figures 2A, B**). In contrast, β_3_ ****integrin was expressed by 60-65% of skin-derived Tregs and 40-50% of Tregs from skin dLNs, and inflammation did not alter expression levels in either the skin or skin dLNs (**Supplementary Figures 2C, D**). These findings indicate that the marked differences in the role of the α_v_ integrin in intradermal Treg migration at different phases of the CS response are not matched by similarly dramatic alterations in expression of this integrin or either of its two main binding partners.

### Dermal Tregs Expressing α_v_ Integrin Display a Highly Activated Phenotype

**Figure 7:**
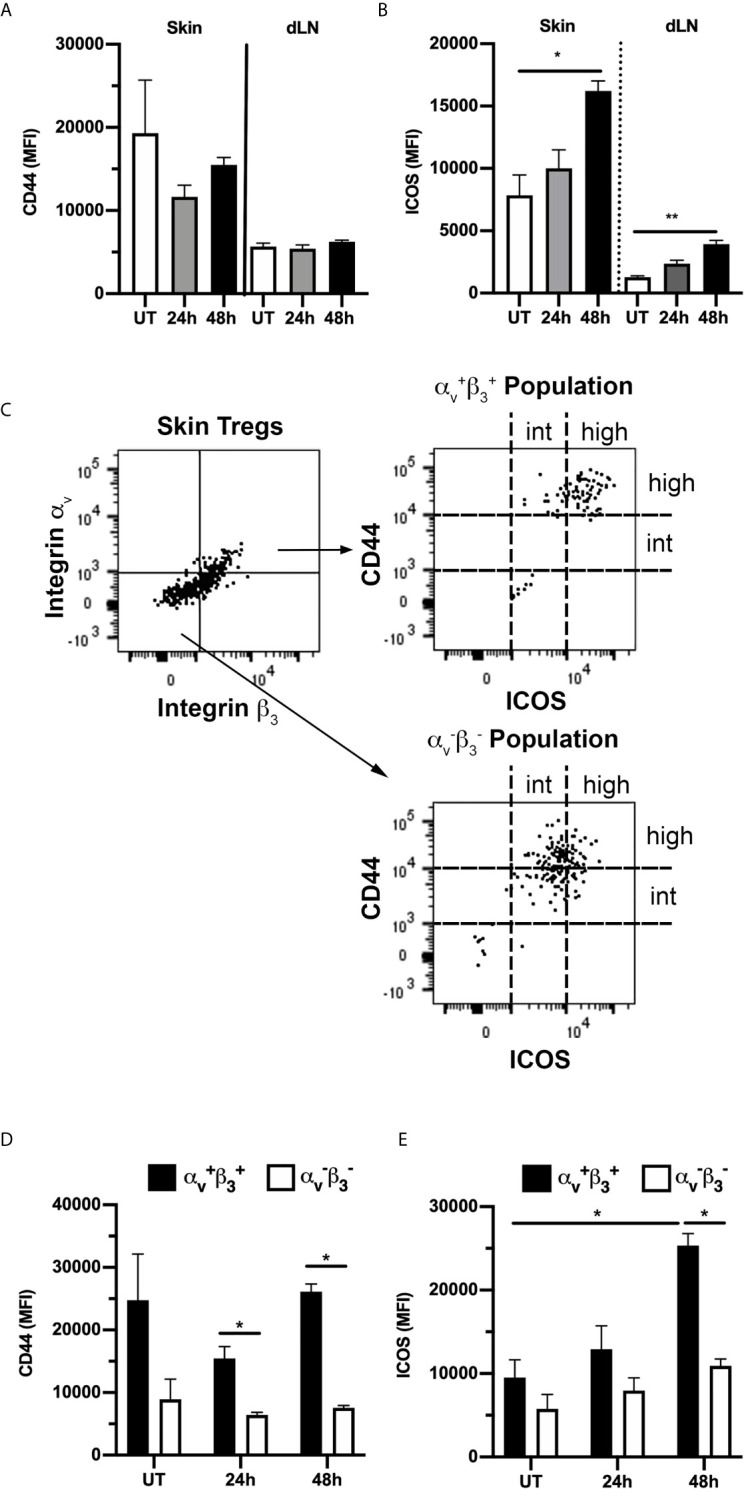
α_v_β_3_^+^ Tregs in skin have a highly-activated phenotype. Expression of activation markers CD44 and ICOS on Tregs from skin and draining lymph nodes (dLN) of untreated (UT) mice, and 24 and 48 h post CS induction was assessed using flow cytometry. **(A, B)** Expression (MFI) of CD44 **(A)** and ICOS **(B)** on all Tregs, shown for both skin and dLN-derived cells in UT mice, and 24 and 48 h post CS-induction. **(C–E)** Comparison of expression of CD44 and ICOS on α_v_^+^β_3_^+^ and α_v_^-^β_3_^-^ Tregs. **(C)** Representative flow cytometry dot plots showing gating used to define α_v_^+^β_3_^+^ and α_v_^-^β_3_^-^ Treg populations (left panel) from skin Tregs of an UT mouse, and CD44/ICOS expression on the two populations (right hand panels). **(D, E)** Quantitation of CD44 **(D)** and ICOS **(E)** expression (MFI) on α_v_^+^β_3_^+^ and α_v_^-^β_3_^-^ skin Tregs from UT mice, and 24 and 48 h post CS induction. Data are shown as mean ± SEM derived from 4 mice/group. Data were evaluated via Kruskal-Wallis test with Dunn’s multiple comparison tests between all timepoints. *p < 0.05, **p < 0.01 for the comparisons shown.

Skin Tregs have previously been reported to display a distinct phenotype, with high expression of activation markers such as CD44 and ICOS as well as a range of adhesion molecules necessary for skin entry (3, 33, 34). This was confirmed here by comparison of CD44 and ICOS expression on dermal Tregs with that on Tregs in the skin dLN (**Figures 7A, B**). In contrast it was apparent from our assessment of Treg integrin expression that expression of RGD-binding integrins is less uniform within this population. Therefore, we next examined the correlation between expression of α_v_ and β_3_ integrins and the activation markers, CD44 and ICOS. Comparison of α_v_^+^β_3_^+^ and α_v_^-^β_3_^-^ Tregs revealed that the α_v_^+^β_3_^+^ population have a particularly highly-activated phenotype with high expression of CD44 and ICOS, while α_v_^-^β_3_^-^ Tregs displayed intermediate levels of these molecules (**Figures 7C–E**). This difference was most apparent for CD44 at 24 and 48 hours, and at 48 hours post CS for ICOS. Notably, of the ~2% of α_v_^+^β_3_^+^ ****Tregs in skin dLN in untreated mice (**Figure 6**), 25% also display the CD44^high^ICOS^high^ phenotype (**Supplementary Figure 3**). Moreover, this percentage increased significantly during inflammation, applying to ~75% of α_v_^+^β_3_^+^ Tregs 48 hours post CS challenge (**Supplementary Figure 3D**). Also, the total numbers of α_v_^+^β_3_^+^- CD44^high^ICOS^high^ Tregs were significantly elevated in dLNs at 48 hours post CS induction compared to UT mice (**Supplementary Figure 3E**), an observation potentially explained by extensive migration of Tregs from inflamed skin to the dLNs (34).

### Dermal Treg Migration in Uninflamed and Inflamed Skin Is Dependent on Signalling Through the PI3K p110δ Subunit

**Figure 8:**
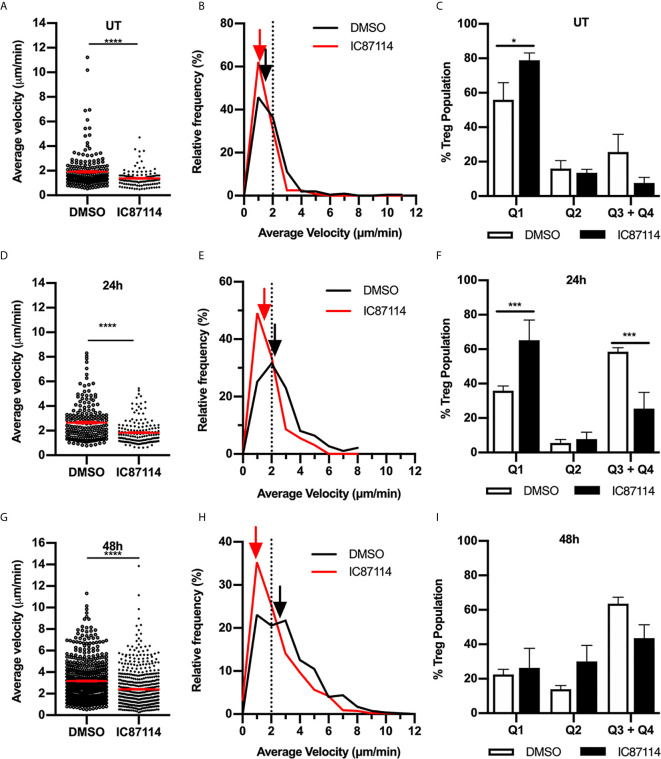
Signalling through the PI3K p110δ subunit promotes Treg migration in resting and inflamed skin. The impact of PI3K p110δ on dermal Treg migration was assessed using MP-IVM, following administration of IC87114 or DMSO vehicle 2 h prior to visualization by MP-IVM. Treg migration was assessed in untreated (UT) skin **(A–C)**, and 24 **(D–F)** and 48 h **(G–I)** post CS challenge. **(A, D, G)** Average velocities of Treg migration. Data show velocities for individual Tregs as well as group mean ± sem. **(B, E, H)** Relative frequency distributions of the average velocities of individual Tregs from IC87114-treated and control mice. Arrows indicate the median velocity, and the dotted line indicates the 2 µm/min threshold of motility. **(C, F, I)** Distributions of Tregs within the four quadrants of the velocity vs. confinement plots shown for UT mice, and 24 and 48 h post CS induction. Data represent the mean ± SEM of > 120 cells (UT) and > 300 cells (CS-challenged mice) derived from 3-7 mice/group. Quadrant data represent mean ± SEM of data averaged from 3 fov/mouse. Data in **(A, D, G)** were analysed by Mann-Whitney test. ****p < 0.0001 for comparisons shown. Data in **(C, F, I)** were analysed using a multiple comparison *t* test using the Holm-Sidak method (alpha 0.05). *p < 0.05, ***p < 0.001 for comparisons shown.

The PI3K subunit p110δ is activated in response to TCR-mediated antigen recognition or co-stimulatory receptor ligation and has been shown to control antigen-induced Treg migration into tissue (17, 24). To investigate the role of p110δ in Treg intradermal migration, we treated Foxp3-GFP mice with the p110δ-selective inhibitor IC87114 (**Figure 8**). In uninflamed mice, p110δ inhibition significantly inhibited the small amount of Treg migration observed in the dermis (**Figures 8A–C**). This was highlighted in the velocity versus confinement plot showing that this treatment led to a significant increase in the percentage of Tregs within the static Q1 quadrant (**Figure 8C**). This effect of p110δ inhibition was more striking when examined 24 hours after induction of the CS response (**Figures 8D–F**, **Supplementary Video 5**). IC87114 induced a significant reduction in average Treg velocity compared to vehicle controls (**Figure 8D**), due to a significant reduction in the proportion of highly-migratory Tregs and a corresponding significant increase in the proportion of static Tregs (**Figures 8E, F**). 48 hours post CS challenge, the effect of IC87114 administration on Treg dermal motility was more subtle in that it again induced a significant reduction in average Treg migration speed (**Figures 8G, H**), although this response occurred in the absence of significant alterations in the proportions of Tregs within different migration quadrants (**Figure 8I**). Interestingly, p110δ inhibition also significantly reduced Treg motility in a model of innate skin inflammation (**Supplementary Figure 4**). Together these findings indicate that signalling through the PI3K p110δ pathway contributes to Treg migration under resting conditions and in both innate and CS-driven responses.

## Discussion

Treg migration into peripheral tissue is critical to their function in controlling local inflammatory responses. What is less well understood is the behaviour of Tregs upon their entry into inflamed peripheral organs. Here we show in a model of antigen-driven skin inflammation, that as inflammation develops, Tregs accumulate in highly dynamic clusters adjacent to hair follicles in the upper dermis. Moreover, Tregs markedly increase their migratory behaviour with elevated migration persisting well after the inflammatory response has resolved (13, 35). The mechanisms supporting this migration differ at different phases of the response. At the peak of inflammation, Tregs migrate via α_v_ integrin-mediated interactions with components of the ECM, with Tregs expressing high levels of this integrin being of a highly-activated effector phenotype. However, 24 hours later, α_v_β_1_ integrin serves to limit Treg migration. In contrast to these divergent results, PI3K p110δ-dependent signalling was critical to migration both under homeostatic conditions and at all stages of inflammation examined. Together these findings demonstrate that control of intradermal Treg migration during contact sensitivity-mediated skin inflammation is multifactorial and dependent on the phase of the inflammatory response under investigation. It remains to be determined whether this is also the case in other forms of skin inflammation, such as that induced during pathogen-driven inflammation.

One of the most striking observations during the CS response was the tendency of Tregs to congregate and dynamically migrate within clusters adjacent to hair follicles, particularly in the later stages of the response when inflammation was resolving. Previous studies of antigen-driven skin inflammation have revealed that immune cells including conventional CD4^+^ T cells, inflammatory monocytes and dendritic cells also congregate in similar structures (29, 36, 37). This raises the intriguing possibility that all of these immune cells are colocalising in these clusters, affording Tregs the opportunity to dynamically interact with these pro-inflammatory subsets and modulate their function. In future studies, this possibility will be investigated in imaging studies using strategies to label additional immune cell subsets in addition to Tregs.

It has been previously shown that conventional CD4^+^ Th1/Th2 effector cells require α_v_/β_1_/β_3_-mediated interactions to move within the skin and exert their effector function (14, 15). Here we observed at the peak of inflammation that Tregs also utilize the α_v_ integrin to migrate within the skin. The finding that Treg migration was also reduced by RGD peptides indicates that the α_v_ integrin was mediating migration via interaction with RGD-containing ECM proteins. However, our evidence indicated that this occurred in the absence of a role for the β_1_ integrin acting alone, despite Tregs expressing high levels of this molecule. As the majority of Tregs also express β_3_, and RGD peptides reduce Treg migration at this time point, this provides evidence that the ECM-binding α_v_β_3_ integrin pairing is a key contributor to Treg migration under these circumstances.

However, in contrast, 48 hours after CS induction, inhibition of α_v_ or β_1_ integrins did not decrease Treg interstitial migration, but induced significant increases in Treg velocity, an observation consistent with integrin:ECM interactions acting to restrict Treg movement at this time point. This alteration in migratory mechanisms during the course of the CS response was not due to marked changes in Treg integrin expression, as dermal Tregs expressed high levels of α_v_, β_1_ and β_3_ integrins throughout the response. This indicates that the extensive Treg migration at later stages in the CS response is independent of the α_v_ integrin. It is important to note that concurrently, the biochemical and cellular composition of the skin is undergoing significant alteration, including reduced collagen density (**Figure 2**), and increased fibronectin content along with extensive immune cell infiltration (14, 32). These alterations introduce numerous additional adhesive ligands with the potential to support Treg migration. In addition, immune cells have been shown to be capable of switching between integrin-dependent and -independent mechanisms for interstitial migration (38), and alternative pathways such as a switch to glycolytic metabolism can also promote Treg migration (39). Together these studies demonstrate the potential molecular complexity of Treg interstitial migration, particularly in inflamed skin.

Previous studies of Treg phenotype have shown that cells with elevated levels of adhesion molecules associated with trafficking to and retention within the skin, such as ligands for P- and E-selectin, and CD103, have greater capacity to suppress inflammatory responses (4, 40, 41). A similar observation has recently been shown to apply to integrin α_2_, although the role of this molecule in Treg trafficking is less clear (42). Here we observed that the sub-population of Tregs in the skin that expresses the highest levels of α_v_β_3_ was also highly positive for activation markers CD44 and ICOS, indicating that these cells are among the most highly activated Tregs present in the skin. As elevated ICOS expression distinguishes Tregs with high suppressive function (43, 44), this provides evidence that α_v_β_3_ serves as an additional adhesion molecule marker of highly-suppressive Tregs in inflamed skin. Notably, as the α_v_ integrin was also critical for Treg migration at the peak of the CS response, this is consistent with this adhesion molecule facilitating migration of highly-functional Tregs throughout the inflamed skin.

Previous studies have demonstrated that highly activated (CD44^hi^) Tregs have a greater capacity to migrate from the skin to the draining lymph node (34). Whether this ability corresponds to a greater capacity of α_v_β_3_^hi^ CD44^hi^ ICOS^hi^ Tregs to migrate within the dermis remains to be determined. The role of the α_v_ integrin in Treg function has previously been examined in other tissues, revealing divergent results (16). Studies of inflammatory responses in the brain and colon have demonstrated that many of the Tregs present in these sites of inflammation highly express abundant α_v_. However, in the brain the α_v_ integrin does not contribute to Treg accumulation or inhibitory function, while in the colon, this integrin can contribute to Treg function under some circumstances (16). These studies demonstrate that α_v_ expression on Tregs does not always correspond to a key functional role for this integrin. This finding is consistent with the present observations which clearly show that the role of the α_v_ integrin differs across different phases of CS. Given this, experiments aimed at investigating the contribution of α_v_ integrin-dependent Treg migration to suppression of skin inflammation will need to be mindful of these phase-dependent differences in α_v_ function.

In contrast to the actions of ECM-binding integrins, blocking PI3K p110δ signalling strongly inhibited Treg migration at all stages of the inflammatory response examined, and in uninflamed skin. The PI3K p110δ pathway is of critical importance to Treg development and function, including in their ability to migrate to inflammatory sites. Fu et al. demonstrated that p110δ-mediated signalling downstream of recognition of antigen presented by activated endothelial cells promoted migration of Tregs across the endothelial barrier and accumulation in the inflamed site (24). Here we show that this pathway is also critical to migration of Tregs after they have crossed the endothelial barrier, not only in inflamed skin, but for the low proportion of migratory Tregs in non-inflamed skin. As the PI3K p110δ pathway acts downstream of the TCR and co-stimulatory molecules such as ICOS and CD28, this suggests that Tregs affected by inhibition of this pathway are responding to their cognate antigen via the TCR (39, 44). Studies in which mature Tregs were rendered deficient in the TCR have demonstrated that Tregs require continuous stimulation via the TCR to maintain suppressor function and a transcriptional profile typical of mature, effector Tregs (45, 46). Here, we showed that interfering with signalling downstream of the TCR, as achieved via PI3K p110δ inhibition, also has an effect on Treg behavioural phenotype, rapidly down-regulating migratory function. Whether the effects on Tregs observed in these two studies are due to related mechanisms requires further investigation. However, it should be noted that the experiments in the present study only examined responses within hours of PI3K p110δ inhibition, whereas the work examining the effect of TCR deficiency examined effects over several days.

One question that remains unanswered is the importance of intradermal migration for the anti-inflammatory function of Tregs in the skin. Given that many of the regulatory mechanisms used by Tregs require either cell-cell contact or proximity to the target cell, it is reasonable to hypothesise that the capacity of Tregs to extensively migrate within the skin is critical to their local anti-inflammatory function (47–49). Evidence supporting this idea has come from imaging studies of Tregs in lymph nodes, where they have been observed to undergo direct contact with dendritic cells reducing their capacity to stimulate immune cell activation (50–52). Whether Treg migration serves a similar function in skin remains to be determined. Moreover, Treg migration is also important for the movement of Tregs out of the skin into skin-draining lymph nodes (dLN), a behaviour which is markedly increased during inflammation (34, 53). As shown here and previously, Tregs that enter skin-dLN have an activated memory phenotype and greater suppressive function as compared to Tregs in the LNs under steady-state conditions (8, 34, 42, 53). This recirculation of Tregs from the skin into dLN and beyond into the circulation is believed to further facilitate regulation of skin inflammation. Together these observations support the idea that the capacity of Tregs to undergo interstitial migration is of key importance in their suppressive function as it affects skin inflammation.

In summary these experiments reveal that control of Treg migration in uninflamed skin and skin undergoing a contact sensitivity-mediated inflammatory response is multifactorial and context-dependent. While PI3K p110δ-transduced signals promote Treg migratory function in both resting and inflamed conditions, during inflammation, the ECM-binding α_v_ integrin first promotes and then retards Treg migration at differing phases of the response. How these varying mechanisms of Treg migration within the skin contribute to control of cutaneous inflammatory responses will require further investigation.

## Data Availability Statement

The raw data supporting the conclusions of this article will be made available by the authors, without undue reservation.

## Ethics Statement

The animal study was reviewed and approved by Monash Medical Centre ‘B’ Animal Ethics Committee.

## Author Contributions

MN designed and performed research, analysed data, and wrote paper. ZC and SS designed and performed research, and analysed data. PP performed research and analysed data. MH designed research and wrote paper. All authors contributed to the article and approved the submitted version.

## Funding

This work was supported by the National Health and Medical Research Council (NHMRC), Australia (Senior Research Fellowship IDs 1042775 & 1135971) to MH.

## Conflict of Interest

The authors declare that the research was conducted in the absence of any commercial or financial relationships that could be construed as a potential conflict of interest.
