# Supplementary material for: Dynamic Regulation of the Molecular Mechanisms of Regulatory T Cell Migration in Inflamed Skin
Source: Front Immunol. 2021 May 10;12:655499. doi: 10.3389/fimmu.2021.655499 (PMC8143438; doi:10.3389/fimmu.2021.655499)
Supplement: Supplementary file 1 [file DataSheet_1.pdf]

Supplementary Figures

Norman *et al*.

Dynamic regulation of the molecular mechanisms of regulatory T cell migration in inflamed skin

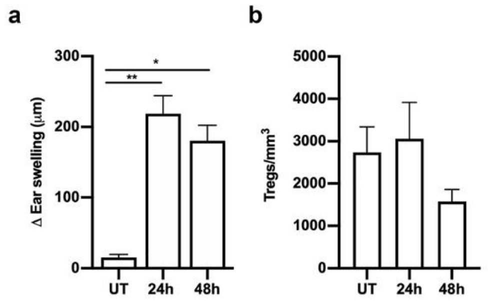

Supplementary Figure 1: Croton oil-induced inflammation does not increase Treg accumulation in the skin. (a) Ear swelling in untreated mice and 24 and 48 h after Croton oil (CO) treatment. (**b**) Quantitation of Treg abundance in the flank dermis of untreated or CO-treated Foxp3-GFP mice, analyzed from MP-IVM recordings. Data represent mean ± SEM of the number of Tregs, expressed as cells/mm^3 dermis, in at least two fov from n=5-8 mice per group. Data were analyzed by Kruskal Wallis test with Dunn's multiple comparison tests between all timepoints. **p*<0.05, ***p*<0.01 for comparisons shown.

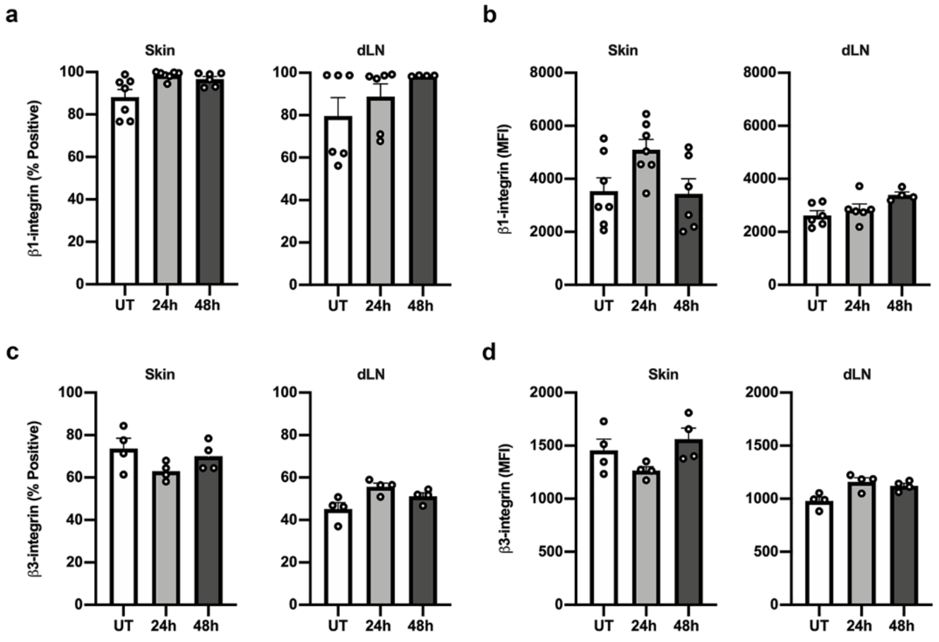

Supplementary Figure 2: β$_1$ and β$_3$ integrin expression on Tregs from skin and dLN. Flow cytometry assessment of surface expression of β$_1$ and β$_3$ integrin on Tregs from skin and dLN of untreated (UT) mice, and mice 24 h and 48 h post CS challenge. Data are shown for % of Tregs positive for β$_1$ **(a)** and β$_3$ **(c)** and MFI of β$_1$ **(b)** and β$_3$ **(d)** expressed on Tregs from skin and dLNs during the CS response. Data are shown as mean ± SEM derived from n=4-7 mice/group.

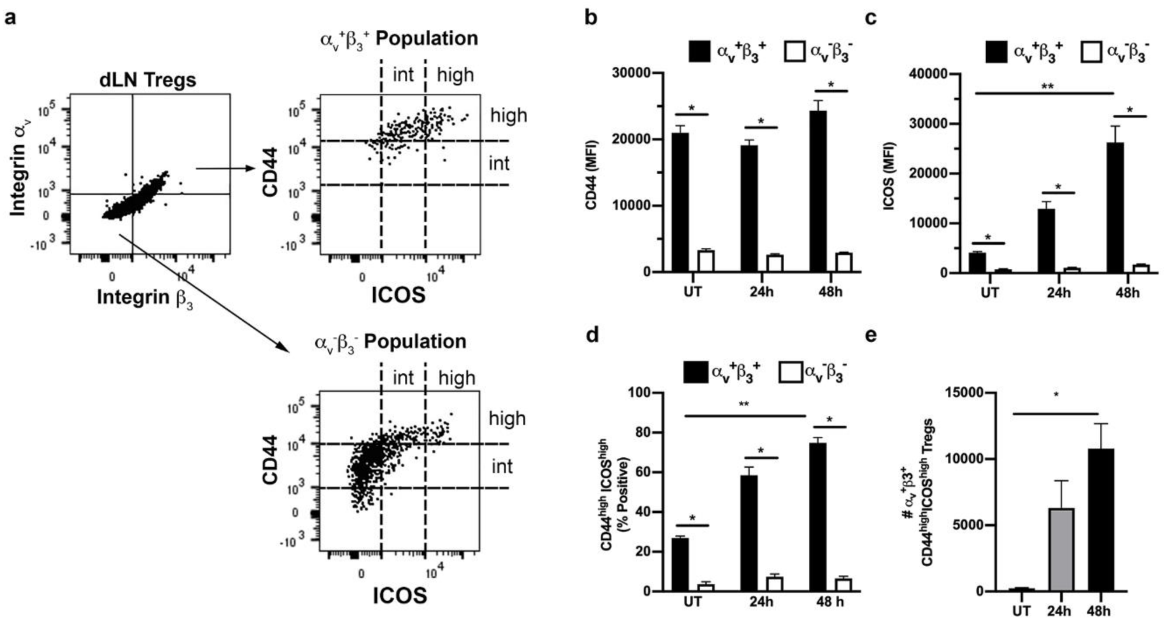

Supplementary Figure 3: Activated $\alpha_v^+\beta_3^+$ Tregs accumulate in skin-draining lymph nodes during inflammation. Expression of integrins and activation markers on dLN Tregs was assessed in untreated (UT) mice, and 24 and 48 h post CS induction using flow cytometry. **(a)** Representative flow cytometry dot blots showing gating used to define $\alpha_v^+\beta_3^+$ and $\alpha_v^-\beta_3^-$ Treg populations (left panel) from dLN from an UT mouse, and CD44/ICOS expression on the two populations (right hand panels). **(b, c)** Quantitation of CD44 **(b)** and ICOS **(c)** expression (MFI) on $\alpha_v^+\beta_3^+$ and $\alpha_v^-\beta_3^-$ dLN Tregs from UT mice and 24 and 48 h post CS induction. **(d)** Proportion of dLN Tregs displaying the CD44^{high}ICOS^{high} phenotype within the $\alpha_v^+\beta_3^+$ and $\alpha_v^-\beta_3^-$ Treg populations, in UT mice, and 24 and 48 h post CS induction. **(e)** Total number of $\alpha_v^+\beta_3^+$ Tregs displaying the CD44^{high}ICOS^{high} phenotype in dLNs from UT mice and 24 h and 48 h post-CS induction. Data in **b-e** are shown as mean ± SEM derived from 4 mice/group. Data were evaluated via Kruskal-Wallis test with Dunn's multiple comparison tests between all timepoints. *p<0.05, **p<0.01 for the comparisons shown.

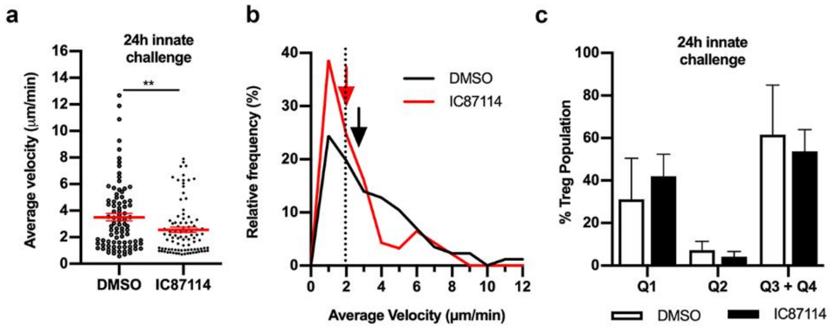

Supplementary Figure 4: Inhibition of signalling through the PI3K p110δ subunit attenuates Treg migration during innate inflammation. Dermal Treg migration was investigated by MP-IVM in a model of innate inflammation induced by application of 1% oxazolone, 24 h post-inflammation induction. The role of PI3K p110δ was assessed by administration of IC87114 (or DMSO vehicle) 2 h prior to visualization. **(a)** Average velocity of Treg migration. **(b)** Frequency distribution of average velocity profile from individual Treg tracks. Arrows indicate the median velocity and the dotted line indicates the 2 μm/min threshold of motility. **(c)** Quadrant profile from the average velocity versus confinement plots of the Treg populations from 24 h oxazolone-challenged mice. Data represent the mean ± SEM of > 90 cells from 3-4 mice/group. Quadrant data represent mean ± SEM of data averaged from 3 fov/mouse. Data analyzed by Mann-Whitney test. ***p*<0.01 for comparison shown.
