# Supplementary material for: Dynamic Regulation of the Molecular Mechanisms of Regulatory T Cell Migration in Inflamed Skin
Source: Front Immunol. 2021 May 10;12:655499. doi: 10.3389/fimmu.2021.655499 (PMC8143438; doi:10.3389/fimmu.2021.655499)
Supplement: Supplementary file 2 [file DataSheet_2.pdf]

Supplementary Video descriptions

Norman *et al*.

Dynamic regulation of the molecular mechanisms of regulatory T cell migration in inflamed skin

Supplementary Video 1: Alterations in intradermal regulatory T cell migration during contact sensitivity. Sequence of videos from multiphoton intravital microscopy (MP-IVM) experiments in Foxp3-GFP mice showing regulatory T cells (Tregs – **green**) in the dermis of untreated skin, and 24 h, 48 h, 72 h and 6 d after induction of contact sensitivity response. Dermal collagen (**blue**) is visible via second harmonic generation (SHG). Each sequence represents ~ 30 min observation time. Yellow – hair follicles. Scale bar = 40 μm.

Supplementary Video 2: Alterations in intradermal regulatory T cell migration during contact sensitivity, with Treg migration paths. Sequence of videos from MP-IVM experiments in Foxp3-GFP mice showing Tregs (**green**) in the dermis of untreated skin, and 24 h, 48 h, 72 h and 6 d after induction of contact sensitivity. Tracks showing migration paths of migratory Tregs have been included. Dermal collagen (**blue**) is visible via second harmonic generation (SHG). Each sequence represents ~ 30 min observation time. Yellow – hair follicles. Scale bar = 40 μm.

Supplementary Video 3: Effect of RGD peptide on intradermal Treg migration in contact sensitivity. Videos from MP-IVM experiments in which Foxp3-GFP mice (Tregs – **green**) were treated with either control (RAD) peptide (left panel) or RGD peptide (right panel), prior to imaging 24 & 48 h after induction of contact sensitivity. Treg migration paths are included. Dermal collagen (**blue**) is visible via second harmonic generation (SHG). Each sequence represents ~ 30 min observation time. Yellow – hair follicles. Scale bar = 40 μm.

Supplementary Video 4: Effect of α_v integrin inhibition on intradermal Treg migration in contact sensitivity. Videos from MP-IVM experiments in which Foxp3-GFP mice (Tregs – **green**) were treated with either isotype control antibody (left panel) or anti-α_v integrin (right panel), prior to imaging 24 & 48 h after induction of contact sensitivity. Treg migration paths are included. Dermal collagen (**blue**) is visible via second harmonic generation (SHG). Each sequence represents ~ 30 min observation time. Yellow – hair follicles. Scale bar = 40 μm.

Supplementary Video 5: Effect of PI3K p110δ inhibition on intradermal Treg migration in contact sensitivity. Videos from MP-IVM experiments in which Foxp3-GFP mice (Tregs – **green**) were treated with either vehicle control (left panel) or PI3K p110δ inhibitor IC87114 (right panel), prior to imaging 24 h after induction of contact sensitivity. Treg migration paths are included. Dermal collagen (**blue**) is visible via second harmonic generation (SHG). Each sequence represents ~ 30 min observation time. Yellow – hair follicles. Scale bar = 40 μm.
